# Evaluation of QuickMIC system for rapid antimicrobial susceptibility testing of Gram-negative pathogens from positive blood cultures, including strains producing extended-spectrum β-lactamases and carbapenemases

**DOI:** 10.1128/spectrum.00370-25

**Published:** 2025-06-10

**Authors:** Fabio Morecchiato, Chiara Chilleri, Alberto Antonelli, Christer Malmberg, Tommaso Giani, Gian Maria Rossolini

**Affiliations:** 1 Department of Experimental and Clinical Medicine, University of Florence 9300, Florence, Tuscany, Italy; 2 Microbiology and Virology Unit, Careggi University Hospital 18561 , Florence, Tuscany, Italy; 3 Gradientech AB - Uppsala (Sweden) 481813 , Uppsala, Uppsala County, Sweden; 4 Department of Medical Sciences, Uppsala University - Uppsala (Sweden) 214437, Uppsala, Uppsala County, Sweden; Johns Hopkins University, Baltimore, Maryland, USA

**Keywords:** bloodstream infections, diagnosis, blood cultures, antimicrobial susceptibility testing, rapid phenotypic antibiogram

## Abstract

The accuracy and turnaround time (TAT) of the QuickMIC system (QMIC) for rapid antimicrobial susceptibility testing (AST) of Gram-negative pathogens from positive blood cultures were evaluated, in comparison with a standard-of-care workflow based on culture and AST with broth microdilution. Essential agreement (EA) and bias were evaluated according to the ISO 20776-2:2021, category agreement (CA), categorical overestimation (CO), and underestimation (CU) rates were evaluated according to the European Committee on Antimicrobial Susceptibility Testing(EUCAST) breakpoints, and major discrepancy (MD) and very major discrepancy (VMD) rates were evaluated according to the Food and Drug Administration (FDA) guidelines and breakpoints. QMIC yielded evaluable data for 103/118 blood cultures (87.3%), including Enterobacterales (*N* = 82), *Pseudomonas aeruginosa* (*N* = 16), and *Acinetobacter baumannii* complex (*N* = 5). Overall, 41 isolates (39.8%) were positive for extended-spectrum β-lactamases and/or carbapenemases. QMIC exhibited EA and CA values of 89.5% and 93.3%, respectively, with values <90% were reported for cefepime, meropenem, and piperacillin-tazobactam. Mean bias was −23.1%, with values >±30% observed with colistin and piperacillin-tazobactam. Overall, CO and CU were observed in 2.3% and 4.4% of cases. MD and VMD with FDA breakpoints were observed in 1.5% and 8.9% of cases, respectively. The lowest accuracy of QMIC in terms of CU was observed with cefepime, colistin, and gentamicin and in terms of VMD with ceftazidime-avibactam, meropenem, and piperacillin-tazobactam. The mean TAT for QMIC was 192 ± 18 min. QMIC demonstrated the potential for rapid AST, with a mean time to result of less than 4 hours, and an overall acceptable accuracy, except with some β-lactams. Further studies are warranted to assess the potential impact of QMIC implementation on clinical outcomes.

**IMPORTANCE:**

The introduction of rapid antimicrobial susceptibility testing (AST) systems for positive blood cultures (BCs) is one of the most promising technological advancements to improve bloodstream infections diagnosis, by providing faster results useful for antimicrobial stewardship. The value of such systems is expected to be higher in settings of high endemicity of antimicrobial resistance, where phenotype prediction is more difficult, and rates of inaccurate empirical therapies are expected to be higher. In this study, we investigated the accuracy and turnaround time (TAT) of a new rapid AST system for positive BCs, in comparison with a standard-of-care workflow based on broth microdilution, including several cases positive for extended-spectrum β-lactamase- and/or carbapenemase-producing Gram negatives. TAT was 192 ± 18 min, and a good accuracy was observed for testing some agents (amikacin, gentamicin, ciprofloxacin, colistin, and ceftazidime-avibactam) but a lower accuracy for testing other agents (cefepime, meropenem, and piperacillin-tazobactam). Further assessment of this technology, after improving accuracy, is warranted to evaluate the clinical impact.

## INTRODUCTION

Bloodstream infections (BSIs) are a major cause of mortality and morbidity worldwide (1, 2). Blood culture (BC) remains the reference approach for the microbiological diagnosis of BSIs (2). However, isolation of infecting pathogen(s) from blood followed by antimicrobial susceptibility testing (AST) requires a relatively long turnaround time (TAT) (often longer than 2–3 days), during which patients are treated with empiric antimicrobial therapy. This entails the risk of using unnecessary broad-spectrum antimicrobials or ineffective antimicrobials, with implications in terms of clinical outcomes, resistance selection, toxicity, and costs (3–7). The risk of prescribing inappropriate empiric therapy is higher in settings affected by high antimicrobial resistance rates, where the prediction of antimicrobial susceptibility profiles of the putative infecting pathogens is more difficult (8).

Two main approaches have recently been developed to reduce the TAT of the BC workflow, including: (i) syndromic panels for rapid molecular detection of the most common pathogens and the most relevant antimicrobial resistance markers from positive BCs and (ii) systems for rapid phenotypic AST from positive BCs. The latter systems are based on disk diffusion (e.g., the European Committee on Antimicrobial Susceptibility Testing [EUCAST]-validated Rapid Antimicrobial Susceptibility Testing [RAST]) (9) or on various technologies (e.g., time-lapse microscopy, metabolomics, flow cytometry, and light scattering) (10–14), which measure the ability of antimicrobials to interfere with bacterial growth at short intervals and translate the results into MIC values. Compared to molecular syndromic panels, the systems for rapid phenotypic AST are somewhat slower but yield results that can be interpreted with established clinical breakpoints and are not challenged by the potential variability of genotypic/phenotypic correlations that can affect the predictivity of the genotypic-based assays (9–14).

QuickMIC (QMIC) (Gradientech AB, Uppsala, Sweden) is a novel commercial system for rapid phenotypic AST of Gram-negative pathogens from positive BCs, based on a microfluidic system able to create an antibiotic gradient in a solid medium, in which bacteria from BC are directly inoculated. Bacterial growth is then automatically monitored by time-lapse light-scatter cytometry, providing AST results within 4 hours (15, 16). As such, QMIC is faster than many other systems for rapid phenotypic AST from positive BCs, which return results after 6–7 hours (15, 16). QMIC is currently validated for *in vitro* diagnostic use with Enterobacterales (including *Klebsiella pneumoniae*, *Escherichia coli*, *Proteus* spp., *Serratia marcescens,* and *Citrobacter* spp.) and Gram-negative non-fermenters (including *Pseudomonas aeruginosa* and *Acinetobacter baumannii* complex) and a panel of antibiotics, including β-lactams (cefepime [CEP], cefotaxime, ceftazidime [CFZ], ceftazidime-avibactam [CAA], meropenem [MER], and piperacillin-tazobactam [PIT]), aminoglycosides (amikacin [AMK], gentamicin [GEN], and tobramycin), tetracyclines (tigecycline [TGC]), fluoroquinolones (ciprofloxacin [CIP]), and colistin (COL) (16).

Although previously evaluated with spiked samples and with real positive BCs, knowledge on the performance of QMIC with multidrug-resistant pathogens is still limited (15, 16). The aim of this study was to evaluate the performance of QMIC, in terms of accuracy and TAT, for rapid AST from positive BCs, also including several isolates producing extended-spectrum β-lactamases (ESBLs) and/or carbapenemases.

## MATERIALS AND METHODS

### Study design

Non-duplicated residual positive BCs (either aerobic or anaerobic vials, whichever flagged positive first), which had tested positive for Gram-negative bacteria by microscopy, were prospectively collected for testing in parallel with the QMIC device and a standard-of-care (SoC) workflow. Samples were included during the period between February and June 2023. To enrich the collection with isolates positive for ESBLs and/or carbapenemases, the BCs selection process also considered preliminary positive results for the presence of these resistance mechanisms after testing with a molecular syndromic panel (FilmArray BCID2, bioMérieux, Marcy l'Etoile, France) to achieve a representativity of at least 15% of these isolates. All BCs had been processed with the BD BACTEC FX blood culture system (Becton Dickinson, Baltimore, MD, USA) and were tested within 24 hours from flagging positive.

Since the study was performed with anonymized residual bacterial cultures and did not include any retrospective evaluation of clinical data nor did foresee any intervention, ethical approval was not required.

### Species identification and AST by the SoC workflow

In the SoC workflow, 10 µL of the positive BC was plated onto chocolate + PolyViteX medium (Liofilchem, Roseto degli Abruzzi, Italy) and incubated for 18–22 hours at 35°C ± 1°C under aerobic conditions. Bacterial identification was then performed using MALDI Biotyper (Bruker, Billerica, MA, USA.) from isolated colonies, and AST was carried out using the MICRONAUT broth microdilution (BMD) system (ITGN plates, Cat. no. 42-E1-184-100, Merlin Diagnostika GmBH, Bornheim, Germany), which is compliant with the ISO 20776-1:2019 standard in terms of inoculum size and testing volumes, according to manufacturer’s instructions. Results were visually read after 18 ± 2 hours of incubation at 35°C ± 1°C in aerobic atmosphere. AST results were interpreted according to EUCAST clinical breakpoints v. 15.0, 2025 (https://www.eucast.org/clinical_breakpoints) and FDA clinical breakpoints (https://www.fda.gov/drugs/development-resources/antibacterial-susceptibility-test-interpretive-criteria).

### AST by QMIC

QMIC was performed from the same vial used with the SoC workflow following manufacturer’s instructions. Briefly, 10 µL of positive BC was mixed with the agar medium provided by the manufacturer, which had previously been melted and equilibrated at 38°C. Meanwhile, the QMIC cassette was filled with the growth medium (Mueller-Hinton broth) provided with the test. The mixture of agar and BC was then filtered with a 3 µm filter (included with QMIC consumables) and loaded into the cassette, which was then cooled at 4°C for 15 min, before loading onto the instrument. Real-time results for each antimicrobial were reported every 10 min for up to 4 hours when definitive results were available for all tested antimicrobials. MIC values obtained via the gradient concentration method were automatically converted to the upper twofold dilution values by the QMIC software (v. 1.0.386.172) and interpreted retrospectively according to EUCAST clinical breakpoints v. 15.0, 2025 (https://www.eucast.org/clinical_breakpoints) or according to the FDA clinical breakpoints based on microbial identification returned by the SoC workflow.

### Comparative analysis of AST results

Comparison of AST results was performed for 10 antimicrobials tested by QMIC, including amikacin (range 1–20 mg/L), cefepime (range 0.5–10 mg/L), ciprofloxacin (range 0.125–2.5 mg/L), colistin (range 0.5–5 mg/L), ceftazidime-avibactam (range 0.5/4–10/4 mg/L), ceftazidime (range 0.5–10 mg/L), gentamicin (range 0.5–10 mg/L), meropenem (range 0.5–10 mg/L), piperacillin-tazobactam (range 2/4–40/4 mg/L), and tigecycline (range 0.06–1.25 mg/L) (16). Cefotaxime and tobramycin were excluded from analysis since they were not tested in the SoC workflow.

For comparison of AST results, percentages of essential agreement (EA) and bias (the latter only for antimicrobials with at least 10 on-scale isolates) were evaluated according to the ISO 20776-2:2021 standard (https://www.iso.org/standard/79377.html), following interpretation of results with EUCAST breakpoints. Categorical agreement (CA), category overestimation (CO), and category underestimation (CU) rates were also evaluated according to EUCAST breakpoints. CO was defined when QMIC reported I instead of an S or R instead of an S or an I; CU was defined when QMIC reported I instead of an R or S instead of an I or an R. As such, CO can lead to an unnecessarily high-dose therapy or to the exclusion of a potentially active antimicrobial, while CU can result in underdosing an antibiotic or using an ineffective agent. CA and EA rates ≥90% and bias rates ≤±30% were considered acceptable. An acceptability threshold of ≤3% for both CO and CU was arbitrarily adopted (11, 17), considering the acceptable threshold used for major discrepancies (MD) and very major discrepancies (VMD) previously indicated by the ISO 20776-2:2007 standard (https://www.iso.org/standard/41631.html). MD and VMD rates were also evaluated according to FDA guidelines following interpretations of results with FDA breakpoints (18).

### TAT analysis

TAT for the QMIC system was obtained by retrieving data automatically saved by the analysis software. Since a hardware device substitution (not related to any aspect of the analysis process) was required after starting the study, TAT for the QMIC systems was available with only a subset of 90 processed samples.

### Characterization of resistance mechanisms

Whenever resistance phenotypes suggestive of ESBL or carbapenemase production were reported by QMIC and/or by SoC workflow results, the presence of several β-lactamase genes was assessed in the isolates obtained from the SoC workflow. The *bla*_CTX-M_, *bla*_OXA-48-like_, *bla*_NDM_, *bla*_VIM_, *bla*_KPC_, and *bla*_IMP_β-lactamase genes were searched using the Allplex Entero-DR Assay (Seegene Inc., Seoul, Republic of Korea). The *bla*_FIM_, *bla*_OXA-23-like_, *bla*_OXA-24_, *bla*_OXA-58_, *bla*_PER_, *bla*_GES_, and *bla*_VEB_β-lactamase genes were searched using an in-house PCR following previously described protocols (19–21). In case of discrepancies between AST results and results expected by molecular testing, Sanger sequencing or WGS analysis with Illumina technology was carried out as previously described (22).

## RESULTS

### Features of the BCs analyzed with the QMIC system and the SoC workflow

A total of 125 BCs positive for Gram-negative bacteria by microscopy were collected for testing in parallel with the QMIC system and the SoC workflow. Seven samples were retrospectively excluded due to the presence of pathogens not validated with QMIC (*N* = 4) or for being polymicrobial but not detected as such by microscopy (*N* = 3). Moreover, technical issues (absence of growth control, cartridge failure, flow generation failure, and/or images acquisition failure) preventing completion of the analytical workflow were reported by QMIC with 38 samples, leading to failure of the entire test (*N* = 15, failure ratio, 12.7%) or to data exclusion for one (*N* = 20) or more (*N* = 3) antimicrobial agents. Therefore, comparative analysis could be performed with 103 positive BCs and a total of 893 antibiotic-bacterium combinations (Table 1).

**TABLE 1:** Acquired ESBL and carbapenemase genes detected in the collection of Gram-negative bacteria^*a*^

		ESBL/carbapenemase genes						
Species	*bla*_CTX-M_	*bla*_KPC_; *bla*_CTX-M_	*bla*_KPC_	*bla*_OXA-23-like_	*bla*_NDM_; *bla*_CTX-M_	*bla*_VEB_	*bla*_FIM_	Total
*Klebsiella pneumonia*	11	8	7	7	1	NI	NI	27
*Escherichia coli*	6	–	–	–	–	NI	NI	6
*Proteus mirabilis*	–	–	–	–	–	1	NI	1
*Enterobacter cloacae* complex	1	–	–	–	–	NI	NI	1
*Pseudomonas aeruginosa*	–	–	–	–	–	NI	1	1
*Acinetobacter baumannii* complex	–	–	–	–	–	NI	NI	5
Total	18	8	7	7	1	1	1	41

^*a*^ –, not detected; NI, not investigated.

According to the SoC workflow, the species from the 103 evaluable samples were *Klebsiella pneumoniae* (*N* = 35, 34%), *Escherichia coli* (*N* = 33, 32%), *Pseudomonas aeruginosa* (*N* = 16, 15.5%), *Acinetobacter baumannii complex* (*N* = 5, 4.8%), and other Enterobacterales (*N* = 14, 13.6%) including *Klebsiella aerogenes*, *Enterobacter cloacae* complex, *Klebsiella oxytoca*, *Proteus mirabilis*, *Morganella morganii*, *Proteus vulgaris*, *Serratia marcescens, Citrobacter freundii,* and *Citrobacter koseri* (Table 1; Table S1). The 103 isolates showed variable resistance profiles and mechanisms. Among evaluable Enterobacterales, the highest resistance rates (>25%) were observed for ciprofloxacin, expanded-spectrum cephalosporins, and piperacillin-tazobactam, with lower resistance rates observed for meropenem (7.3%) and resistance to colistin and ceftazidime-avibactam only observed in a few *K. pneumoniae* isolates. Among *P. aeruginosa*, the highest resistance rates (>25%) were observed for cefepime and piperacillin-tazobactam, with lower resistance rates (<20%) observed for meropenem and other antipseudomonal agents. All the *A. baumannii* complex isolates were resistant to amikacin, ciprofloxacin, gentamicin, and meropenem (Fig. S1). Overall, 19/103 isolates (18%) were positive for an ESBL gene (*bla*_CTX-M_ or *bla*_VEB_), 13 isolates (12%) were positive for a carbapenemase gene (*bla*_KPC_, *bla*_FIM_, or *bla*_OXA-23_), and 9 isolates (8.7%) were positive for a combination thereof (Table 1).

### Comparison of AST results from the QMIC system with those from BMD used in the SoC workflow

A total of 893 results for antibiotic-bacterium combinations obtained with the QMIC systems were evaluable for comparison with those from the SoC workflow.

Overall, comparative analysis showed QMIC EA and CA values of 89.5% and 93.2%, respectively. The EA ranged from 78.4% for piperacillin-tazobactam to 96.9% for tigecycline and amikacin. With β-lactams, EA values <90% were observed, except for ceftazidime-avibactam. The CA values ranged from 84.5% for cefepime and piperacillin-tazobactam to 100% for tigecycline. With β-lactams, CA values <90% were observed with cefepime, meropenem, and piperacillin-tazobactam. The mean bias evaluable with some antibiotics (cefepime, ciprofloxacin, colistin, ceftazidime-avibactam, ceftazidime, meropenem, and piperacillin-tazobactam) was −23.1%, with values exceeding −30% for colistin and piperacillin-tazobactam, respectively. Finally, CO and CU, as defined in the Materials and Methods section, were observed in 2.3% and 4.4% of cases (Table 2), while discrepancies in terms of MD and VMD, as defined by FDA criteria, were observed in 1.5% and 8.9% of cases, respectively (Table S2).

**TABLE 2:** Overall performance of QMIC compared to BMD used in the SoC workflow

	EA (%)	Bias^*a*^ (on-scale isolates)	CA (%)	CO (%)	CU (%)
Total Enterobacterales					
AMK	78/80 (97.5)		79/80 (98.8)	0/80 (0)	1/80 (1.3)
CEP	74/82 (90.2)	**−44.5%** (13)	**72/82** (**87.8**)	1/82 (1.2)	**9/82** (**11.0**)
CIP	77/80 (96.3)	−5.3% (17)	76/80 (95.0)	2/80 (2.5)	2/80 (2.5)
COL	72/75 (96.0)	**−65.7%** (**41**)	74/75 (98.7)	1/75 (1.3)	0/75 (0)
CAA	77/79 (97.5)		79/79 (100)	0/79 (0)	0/79 (0)
CFZ	73/78 (93.6)	−10.2% (26)	75/78 (96.2)	2/78 (2.6)	1/78 (1.3)
GEN	74/79 (93.7)		75/79 (94.9)	**3/79** (**3.8**)	1/79 (1.3)
MER	76/82 (92.7)	**−40.0%** (14)	77/82 (93.9)	0/82 (0)	**5/82** (**6.1**)
PIT	**66/81** (**81.5**)	**−41.0%** (**27**)	**69/81** (**85.2**)	2/81 (2.5)	**10/81** (**12.3**)
TGC^c^	31/32 (96.9)		32/32 (100)	0/32 (0)	0/32 (0)
Total	698/748 (93.3)		708/748 (94.7)	11/748 (1.5)	**29/748** (**3.9**)
*Klebsiella pneumonia*					
AMK	35/35 (100)		35/35 (100)	0/35 (0)	0/35 (0)
CEP	**29/35** (**82.9**)	**−44.8%** (10)	**27/35** (**77.1**)	0/35 (0)	**8/35** (**22.9**)
CIP	33/35 (94.3)	−7.9% (11)	32/35 (91.4)	1/35 (2.9)	**2/35** (**5.7**)
COL	32/35 (91.4)	−75.2% (21)	34/35 (97.1)	1/35 (2.9)	0/35 (0)
CAA	31/33 (93.9)		33/33 (100)	0/33 (0)	0/33 (0)
CFZ	30/33 (90.9)	−1.9% (14)	32/33 (97.0)	0/33 (0)	1/33 (3.0)
GEN	32/34 (94.1)		33/34 (97.1)	1/34 (2.9)	0/34 (0)
MER	**30/35** (**85.7**)	**−40.6%** (13)	**30/35** (**85.7**)	0/30 (0)	**5/30** (**14.3**)
PIT	**27/34** (**79.4**)	−12.3% (12)	**29/34** (**85.3**)	1/34 (2.9)	**4/34** (**11.8**)
Total	279/309 (90.3)		285/309 (92.2)	4/309 (1.3)	**20/309** (**6.5**)
*Escherichia coli*					
AMK	31/31 (100)		31/31 (100)	0/31 (0)	0/31 (0)
CEP	32/33 (97.0)		32/33 (97.0)	0/33 (0)	1/33 (3.0)
CIP	31/31 (100)		31/31 (100)	0/31 (0)	0/31 (0)
COL	32/32 (100)	**−50.2%** (15)	32/32 (100)	0/32 (0)	0/32 (0)
CAA	33/33 (100)		33/33 (100)	0/33 (0)	0/33 (0)
CFZ	32/32 (100)		32/32	0/32 (0)	0/32 (0)
GEN	29/31 (93.5)		29/31 (93.5)	**2/32** (**6.5**)	0/32 (0)
MER	33/33 (100)		33/33 (100)	0/33 (0)	0/33 (0)
PIT	**29/33** (**87.9**)		**29/33** (**87.9**)	0/33 (0)	**4/33** (**12.1**)
TGC	31/32 (96.9)		32/32 (100)	0/32 (0)	0/32 (0)
Total	313/321 (97.5)		314/321 (97.8)	2/321 (0.6)	5/321 (1.6)
Other Enterobacterales*					
AMK	**12/14** (**85.7**)		13/14 (92.9)	0/14 (0)	**1/14** (**7.1**)
CEP	13/14 (92.9)		13/14 (92.9)	**1/14** (**7.1**)	0/14 (0)
CIP	13/14 (92.9)		13/14 (92.9)	**1/14** (**7.1**)	0/14 (0)
COL	8/8 (100)		8/8 (100)	0/8 (0)	0/8 (0)
CAA	13/13 (100)		13/13 (100)	0/13 (0)	0/13 (0)
CFZ	**11/13** (**84.6**)		**11/13** (**84.6**)	**2/13** (**15.4**)	0/13 (0)
GEN	13/14 (92.9)		13/14 (92.9)	0/14 (0)	**1/14** (**7.1**)
MER	13/14 (92.9)		14/14 (100)	0/14 (0)	0/14 (0)
PIT	**10/14** (**71.4**)		**11/14** (**78.6**)	**1/14** (**7.1**)	**2/14** (**14.3**)
Total	**106/118** (**89.8**)		109/118 (92.4)	**5/118** (**4.2**)	**4/118** (**3.4**)
AMK	13/14 (92.9)		13/14 (92.9)	0/14 (0)	**1/14** (**7.1**)
CEP	**6/15** (**40.0**)		**10/15** (**66.7**)	**4/15** (**26.7**)	**1/15** (**6.7**)
CIP	15/16 (93.8)		16/16 (100)	0/16 (0)	0/16 (0)
COL	**10/16** (**62.5**)		**14/16** (**87.5**)	**2/16** (**12.5**)	0/16 (0)
CAA	**9/16** (**56.3**)		15/16 (93.8)	0/16 (0)	**1/16** (**6.3**)
CFZ	**10/16** (**62.5**)		**14/16** (**87.5**)	0/16 (0)	**2/16** (**12.5**)
MER	**9/16** (**56.3**)		**11/16** (**68.8**)	**4/16** (**25.0**)	**1/16** (**6.3**)
PIT	**10/16** (**62.5**)		**13/16** (**81.3**)	0/16 (0)	**3/16** (**7.2**)
Total	**82/125** (**65.6**)		**106/125** (**84.8**)	**10/125** (**8.0**)	**9/125** (**7.2**)
AMK	3/3 (100)		3/3 (100)	0/3 (0)	0/3 (0)
CIP	5/5 (100)		5/5 (100)	0/5 (0)	0/5 (0)
COL	5/5 (100)		5/5 (100)	0/5 (0)	0/5 (0)
GEN	4/4 (100)		4/4 (100)	0/4 (0)	0/4 (0)
MER	**2/3** (**66.7**)		**2/3** (**66.7**)	0/3 (0)	**1/3** (**33.3**)
Total	19/20 (95.0)		19/20 (95.0)	0/20 (0)	**1/20** (**5.0**)
Total collection					
AMK	94/97 (96.9)		95/97 (97.9)	0/97 (0)	2/97 (2.1)
CEP	**80/97** (**82.5**)	−18.6% (24)	**82/97** (**84.5**)	**5/97** (**5.2**)	**10/97** (**10.3**)
CIP	97/101 (96.0)	−4.7% (29)	97/101 (96.0)	2/101 (2.0)	2/101 (2.0)
COL	87/96 (90.6)	**−68.0%** (**58**)	93/96 (96.9)	**3/96** (**3.1**)	0/96 (0)
CAA	86/95 (90.5)	−1.7% (15)	94/95 (98.9)	0/95 (0)	1/95 (1.1)
CFZ	**83/94** (**88.3**)	−7.8% (41)	89/94 (94.7)	2/94 (2.1)	**3/94** (**3.2**)
GEN	78/83 (94.0)		79/83 (95.2)	**3/83** (**3.6**)	1/83 (1.2)
MER	**87/101** (**86.1**)	−28.3% (23)	**90/101** (**89.1**)	**4/101** (**4.0**)	**7/101** (**6.9**)
PIT	**76/97** (**78.4**)	**−32.9%** (**40**)	**82/97** (**84.5**)	2/97 (2.1)	**13/97** (**13.4**)
TGC^*c*^	31/32 (96.9)		32/32 (100)	0/32 (0)	0/32 (0)
Total	**799/893** (**89.5**)		833/893 3)	21/893 (2.3)	**39/893** (**4.4**)

^*a*^ Bias was evaluated when at least 10 on-scale MIC values were available; EA, CA, and CO defined when QMIC reported an I instead of S or an R instead of S/I; CU defined when QMIC reported an I instead of R or an S instead of I/R; out of range values are reported in bold, considering, a 3% threshold for CO, CU, VMD, and MD and ≤±30% for bias. ^c^ Clinical breakpoint of tigecicline are evaluable only for *Escherichia coli* ; * including 3 *Klebsiella aerogenes*, 2 *Enterobacter cloacae complex*, 2 *Klebsiella oxytoca*, 2 *Proteus mirabilis*, 1 *Morganella morgani*i, 1 *Proteus vulgaris*, 1 *Serratia marcescens*, 1 *Citrobacter freundii* and 1 *Citrobacter koseri*.

For Enterobacterales, EA and CA values of QMIC were >90% (93.3% and 94.7%, respectively) considering all tested agents. EA and/or CA values <90% were observed with piperacillin-tazobactam and cefepime. CO values >3% were observed with gentamicin, and CU values >3% were observed with meropenem, cefepime, and piperacillin-tazobactam (Table 2).

For *P. aeruginosa,* EA and CA values of QMIC were <90% (65.6% and 84.8%, respectively) considering all tested agents. EA values >90% were only observed with amikacin and ciprofloxacin, while CA values >90% were observed with the latter agents and ceftazidime-avibactam. CO and CU rates >3% were observed with several agents (Table 2).

For *A. baumannii,* both EA and CA values of QMIC were 95.0% considering all tested agents, and EA and CA values <90% were only observed with meropenem (Table 2).

We encountered a few examples in which the results of QMIC were useful to avoid misleading predictions by molecular testing. Namely, a *K. pneumoniae* positive for the *bla*_NDM_ metallo-β-lactamase and *bla*_CTX-M_ ESBL genes by molecular testing showed an ESBL-like phenotype with QMIC (MICs of 4 and ≤0.5 mg/L for cefepime and meropenem, respectively; ceftazidime MIC was not available due to technical failure) confirmed also by BMD. In this case, the *bla*_NDM-1_ gene carried an insertion of a T nucleotide after position 215, resulting in a premature stop codon at nucleotide 285, causing the uncommon phenotype (14) (Table S3). Moreover, a *K. pneumoniae* positive for the *bla*_KPC_ serine-carbapenemase gene by molecular testing was reported susceptible to meropenem and resistant to ceftazidime-avibactam by QMIC (MICs of 4 and >16 mg/L, respectively). Both BCID2 and PCR reported the presence of the gene, while Sanger sequencing led to the detection of *bla*_KPC-31_ variant (Table S3).

### TAT of the QMIC system

Analysis of the TAT with the QMIC system could be performed with 90 samples (71 Enterobacterales and 19 non-fermenters). Overall, the mean TAT was 192 ± 18 min (range 153–230 min), being slightly longer with non-fermenters (mean 211 ± 16 min, range 170–230 min) than with Enterobacterales (mean 188 ± 15 min, range 153–228 min). Results in less than 3 hours were obtained for amikacin with both Enterobacterales and non-fermenters, and for colistin and ceftazidime-avibactam with Enterobacterales (Fig. S2).

## DISCUSSION

Systems for rapid phenotypic AST implementable in the BC workflow can significantly reduce the TAT, improving antimicrobial stewardship and clinical outcomes of confirmed BSI (10, 13). Among these systems, QMIC is a novel platform based on controlled antimicrobials gradient in a microfluidic array in combination with time-lapse microscopy, which can analyze positive BCs and return AST results within 4 hours (15, 16). Initial experiences with QMIC revealed an overall CA of 96.0%–99.0% when compared to BMD, manual or automated (15, 23, 24). However, experiences with this system remain limited, particularly with multidrug-resistant Gram-negative pathogens, including strains producing ESBLs and carbapenemases, which are known to pose challenges to AST methods.

This study evaluated the QMIC system in comparison with a conventional workflow using a commercial BMD system compliant with the ISO 20776-1:2019 standard concerning inoculum size, broth composition, and testing volume, with a real-life case series of positive BCs by Enterobacterales and Gram-negative non-fermenters, including several isolates producing ESBLs and/or carbapenemases. Although an overall fair correlation was observed between QMIC and BMD in terms of EA and CA, QMIC exhibited a general trend to undercall MIC values, which was more pronounced with some bacterial species and some antibiotics. Altogether, QMIC yielded relatively high rates of CU (as defined in this work) with β-lactam antibiotics (e.g., cefepime, ceftazidime, meropenem, and piperacillin-tazobactam), and high rates of VMD (according to FDA criteria) were registered for all antimicrobials except amikacin and colistin, while high rates of MD were observed with cefepime, gentamicin, and piperacillin-tazobactam. The impact of this overall undercalling trend could be higher when dealing with multidrug-resistant organisms since it could lead to the clinical use of ineffective antimicrobials.

As such, the results of this study were consistent with those of previous studies for Enterobacterales, where lower than acceptable CA and/or EA values were observed with several antimicrobials (15, 16). The lowest accuracy was observed with *P. aeruginosa*, with EA and CA values consistently lower than 90% for various agents (mostly β-lactams), in agreement with previous observations (16). The issue of QMIC performances with *P. aeruginosa* was highlighted by results obtained with the uncommon *bla*_FIM_-positive isolate, for which QMIC reported false susceptibilities to ceftazidime, ceftazidime-avibactam, and meropenem (MIC ≤0.5, ≤0.5, and 8 mg/L, respectively). However, QMIC was able to correctly determine two other unusual profiles among *K. pneumoniae* isolates (Table S3).

Failure to obtain results, either due to technical issues or slow bacterial growth, was observed for at least one antibiotic included on the test in 30.4% of all samples, apparently unrelated to specific patterns, counting for 2.8% of the total evaluable sample/antibiotic combinations. The impact of randomly missing results could negatively affect the potential use in laboratory routine by increasing costs of eventual test repetition and by delaying the revision of empirical therapy. It is worth noting that the QMIC system has only recently reached the market and is rapidly undergoing development focused on increasing stability and yield.

The inclusion of different antimicrobial compounds and/or novel inhibitors (e.g., cefiderocol and meropenem-vaborbactam) in addition to a Gram-positive panel (both currently under development) could represent valuable improvements for QMIC applications in clinical laboratory routine. Results obtained with this study, including a high number of multidrug-resistant isolates, will be of support for future software upgrades (including interpretation of MIC results), and automation possibilities (due to handling procedures required for QMIC setup) could be further advantageous. Interpretation of MIC results according to EUCAST breakpoints and expert rules has been introduced into the QMIC system in the latest update, whereas a device for automated sample preparation is under development (personal communication), reflecting the need to continuously investigate the performance of commercial RAST systems.

RAST methods are, in general, relatively new and represent a significant advancement in clinical microbiology, offering the potential to streamline workflows and provide faster and more actionable results for antimicrobial susceptibility testing. However, as these systems are refined, ongoing evaluations of their accuracy, reliability, and integration into routine clinical practice are essential. Such assessments should include a focus on their capacity to address emerging resistance mechanisms, compatibility with evolving breakpoints, adaptability to various clinical settings, and the addition of data on antimicrobial use, reduction of the time to active treatment, and clinical outcomes to fully evaluate the benefits of fast AST, in terms of either escalating or de-escalating empirical treatment. The continued evolution of RAST systems, including QMIC, underscores their promise in combating antimicrobial resistance while highlighting the importance of rigorous validation to ensure their effectiveness in improving patient outcomes.

## SUPPLEMENTAL MATERIAL

Supplemental material spectrum.00370-25-s0001.docx Tables S1 to S3; Figures S1 and S2.

ASM does not own the copyrights to Supplemental Material that may be linked to, or accessed through, an article. The authors have granted ASM a non-exclusive, world-wide license to publish the Supplemental Material files. Please contact the corresponding author directly for reuse.
