# Supplementary material for: Evaluation of QuickMIC system for rapid antimicrobial susceptibility testing of Gram-negative pathogens from positive blood cultures, including strains producing extended-spectrum β-lactamases and carbapenemases
Source: Microbiol Spectr. 2025 Jun 10;13(7):e00370-25. doi: 10.1128/spectrum.00370-25 (PMC12210996; doi:10.1128/spectrum.00370-25)
Supplement: Supplemental material — Tables S1 to S3; Figures S1 and S2. [file spectrum.00370-25-s0001.docx]

| **Antimicrobial** | **Total**  **tested** | **Reported**  **failures** | **Tests eligible for**  **AST comparison** | **Failure**  **ratio** |
| --- | --- | --- | --- | --- |
| AMK | 103 | 6 | 97 | 5.8% |
| CEP | 98 | 1 | 97 | 1.0% |
| CIP | 103 | 2 | 101 | 1.9% |
| COL | 98 | 2 | 96 | 2.0% |
| CAA | 98 | 3 | 95 | 3.1% |
| CFZ | 98 | 4 | 94 | 4.1% |
| GEN | 87 | 4 | 83 | 4.6% |
| MER | 103 | 2 | 101 | 1.9% |
| PIT | 98 | 1 | 97 | 1.0% |
| TGC | 33 | 1 | 32 | 3.0% |
| Total | 919 | 26 | 893 | 2.8% |

**Table S1.** Summary of technical issues reported by the QMIC system with tested antimicrobial agents in the 102 completed tests. AMK: amikacin; CEP: cefepime; CIP: ciprofloxacin; COL: colistin; CAA: ceftazidime-avibactam; CFZ: ceftazidime; GEN: gentamicin; MER: meropenem; PIT: piperacillin-tazobactam; TGC: tigecycline.

* including 3 *Klebsiella aerogenes*, 2 *Enterobacter cloacae complex*, 2 *Klebsiella oxytoca*, 2 *Proteus mirabilis*, 1 *Morganella morganii*, 1 *Proteus vulgaris*, 1 *Serratia marcescens*, 1 *Citrobacter freundii* and 1 *Citrobacter koseri*.

**Figure S1.** Percentages of susceptibility obtained with BMD-based methodology in isolates for which AST comparison was possible. S: susceptible, standard dosing regimen; I: susceptible, increased exposure; R: resistant, according to EUCAST clinical breakpoints 15.0 (<https://www.eucast.org/clinical_breakpoints>); NA: not applicable. AMK: amikacin; CEP: cefepime; CIP: ciprofloxacin; COL: colistin; CAA: ceftazidime-avibactam; CFZ: ceftazidime; GEN: gentamicin; MER: meropenem; PIT: piperacillin-tazobactam; TGC: tigecycline.

|  | **CA**  **(%)** | **VMD**  **(%)** | **MD**  **(%)** |
| --- | --- | --- | --- |
| **Total collection** |  |  |  |
| **AMK** | 95/95 (100) | 0/8 (0) | 0/92 (0) |
| **CEP** | 79/100 (79) | 1/37 (2.7) | 2/55 (3.6) |
| **CIP** | 94/99 (94.9) | 3/42 (7.3) | 1/55 (1.8) |
| **COL** | 95/97 (97.9) | 0/6 (0) | 1/73 (1.4) |
| **CAA** | 97/98 (99) | 1/2 (50) | 0/96 (0) |
| **CFZ** | 88/96 (91.7) | 3/37 (8.1) | 0/55 (0) |
| **GEN** | 79/81 (97.5) | 1/20 (5) | 3/59 (5.1) |
| **MER** | 75/81 (92.6) | 3/15 (20) | 0/66 (0) |
| **PIT** | 66/80 (82.5) | 5/25 (20) | 2/49 (4.1) |
| **Total** | 768/827 (92.9) | 17/192 (8.9) | 9/600 (1.5) |

**Table S2.** Overall performance of QMIC compared to BMD used in the SoC workflow with FDA breakpoints. CA: categorical agreement; VMD: very major discrepancy; MD: major discrepancy; AMK: amikacin; CEP: cefepime; CIP: ciprofloxacin; COL: colistin; CAA: ceftazidime-avibactam; CFZ: ceftazidime; GEN: gentamicin; MER: meropenem; PIT: piperacillin-tazobactam; Tigecycline was not evaluated due to the restricted range of MIC values tested. Out of range values are reported in red, considering a 3% threshold for MD and a 1.5% threshold for VMD.

|  |  |  | **QuickMIC® MIC (mg/L) [interpretation]** | | | |  |
| --- | --- | --- | --- | --- | --- | --- | --- |
| **Isolate ID** | **Genotype ^a^** | **Expected phenotype** | **CEP** | **CAA** | **CFZ** | **MER** | **Genotype ^b^** |
| FI-GT-Kpn016 | *bla*_NDM_; *bla*_CTX-M_ | Resistance to extended-spectrum β-lactams, carbapenems and β-lactams/β-lactamase inhibitors | 4  [I] | 1  [S] | NA | ≤0.5  [S] | Truncated *bla*_NDM_; *bla*_CTX-M-15_ |
| FI-GT-Kpn060 | *bla*_KPC_ | Resistance to extended-spectrum β-lactams, susceptibility to carbapenems and β-lactams/β-lactamase inhibitors | >16  [R] | >16  [R] | >16  [R] | 4  [I] | *bla*_KPC-31_ |
| ^a^ assessed with BCID2 and PCR.  ^b^ assessed with WGS or Sanger sequencing. | | | | | | | |

**Table S3.** Comparison of expected and obtained phenotypes with genotypes for two *Klebsiella pneumoniae* isolates subjected to WGS analysis or Sanger sequencing. S: susceptible, standard dosing regimen; I: susceptible, increased exposure; R: resistant, according to EUCAST clinical breakpoints 15.0 (https://www.eucast.org/clinical_breakpoints); NA: not available; CEP: cefepime; CAA: ceftazidime-avibactam; CFZ: ceftazidime; MER: meropenem.

**Figure S2.** Mean of time-to-results (TTR) for (from above to bottom) *Enterobacterales*, non-fermenter Gram-negative bacteria (GNB) and total collection. Error bars represent standard deviation.
